# Evaluation of the hepatotoxicity of *Psoralea corylifolia* L. based on a zebrafish model

**DOI:** 10.3389/fphar.2024.1308655

**Published:** 2024-02-21

**Authors:** Shu-Yan Gao, Jing-Cheng Zhao, Qing Xia, Chen Sun, Maimaiti Aili, Ainiwaer Talifu, Shi-Xia Huo, Yun Zhang, Zhi-Jian Li

**Affiliations:** ^1^ Uyghur Medical Hospital of Xinjiang Uyghur Autonomous Region, Ürümqi, China; ^2^ Xinjiang Key Laboratory of Evidence-Based and Translation, Hospital Preparation of Traditional Chinese Medicine, Ürümqi, China; ^3^ College of Pharmacy, Xinjiang Medical University, Ürümqi, China; ^4^ Biology Institute, Qilu University of Technology (Shandong Academy of Sciences), Jinan, China

**Keywords:** *Psoralea corylifolia* L., hepatotoxicity, zebrafish, cholestony, lipid metabolism

## Abstract

**Objective:** *Psoralea corylifolia* L. (FP) has received increasing attention due to its potential hepatotoxicity.

**Methods:** In this study, zebrafish were treated with different concentrations of an aqueous extract of FP (AEFP; 40, 50, or 60 μg/mL), and the hepatotoxic effects of tonicity were determined by the mortality rate, liver morphology, fluorescence area and intensity of the liver, biochemical indices, and pathological tissue staining. The mRNA expression of target genes in the bile acid metabolic signaling pathway and lipid metabolic pathway was detected by qPCR, and the mechanism of toxicity was initially investigated. AEFP (50 μg/mL) was administered in combination with FXR or a peroxisome proliferator-activated receptor α (PPARα) agonist/inhibitor to further define the target of toxicity.

**Results:** Experiments on toxic effects showed that, compared with no treatment, AEFP administration resulted in liver atrophy, a smaller fluorescence area in the liver, and a lower fluorescence intensity (*p* < 0.05); alanine transaminase (ALT), aspartate transaminase (AST), and γ-GT levels were significantly elevated in zebrafish (*p* < 0.01), and TBA, TBIL, total cholesterol (TC), TG, low-density lipoprotein cholesterol (LDL-C), and high-density lipoprotein cholesterol (HDL-C) levels were elevated to different degrees (*p* < 0.05); and increased lipid droplets in the liver appeared as fatty deposits. Molecular biological validation revealed that AEFP inhibited the expression of the FXR gene, causing an increase in the expression of the downstream genes SHP, CYP7A1, CYP8B1, BSEP, MRP2, NTCP, peroxisome proliferator-activated receptor γ (PPARγ), ME-1, SCD-1, lipoprotein lipase (LPL), CPT-1, and CPT-2 and a decrease in the expression of PPARα (*p* < 0.05).

**Conclusion:** This study demonstrated that tonic acid extracts are hepatotoxic to zebrafish through the inhibition of FXR and PPARα expression, thereby causing bile acid and lipid metabolism disorders.

## Graphical Abstract

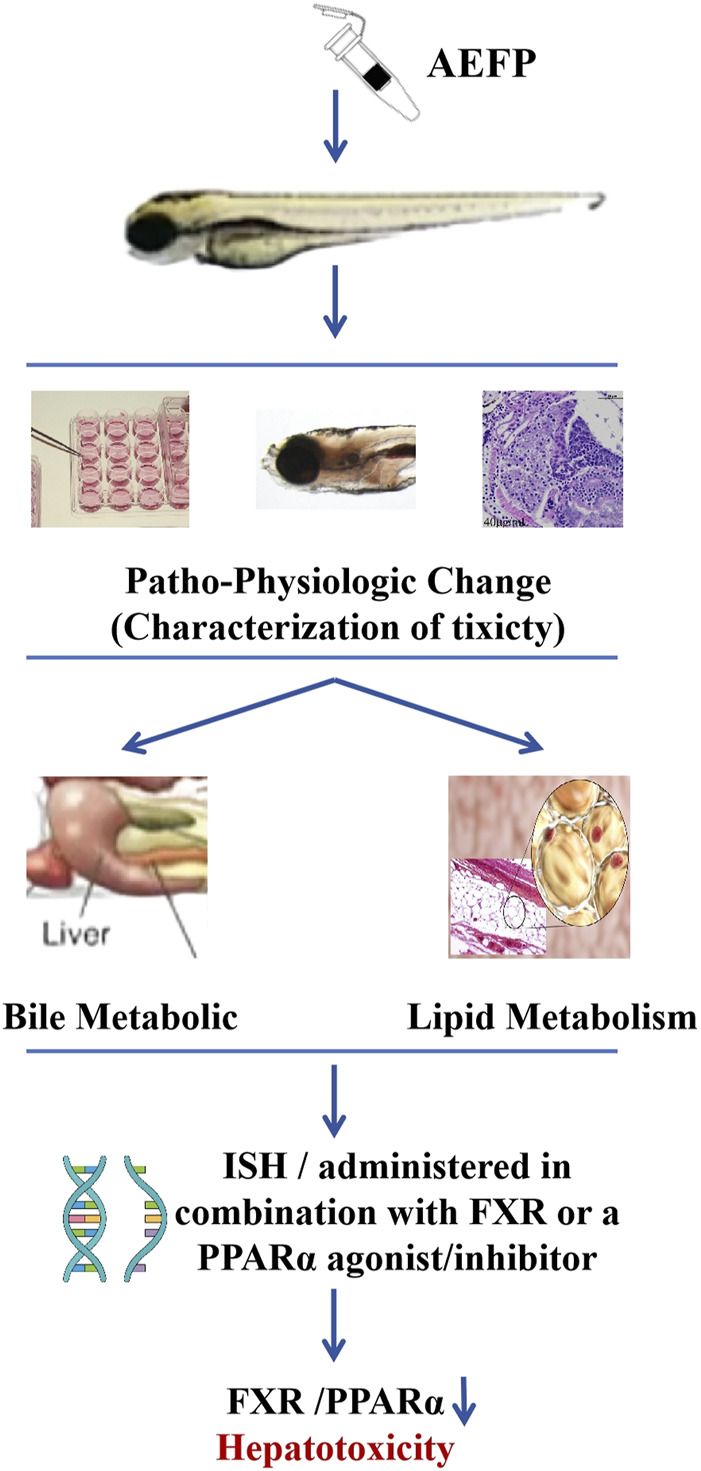

## Introduction

*Psoralea corylifolia* L. (FP), an important traditional herbal medicine, has a long history of clinical application and has been widely used in many countries. The whole plant has important medicinal value and is used to treat various diseases, such as leucoderma, menstrual disorders, uterine bleeding, and endometriosis (Chen et al., 2023; Alam et al., 2018). FP (*Psoralea corylifolia* L. ) and its formulations are also widely used in China for the treatment of bone and skin diseases (Makwana et al., 2020; Li T et al., 2022). More than 200 compounds, mainly coumarins, flavonoids, and terpenoids, have been isolated and identified from psoriasis (Gao et al., 2021; Wu et al., 2020). These major components have biological activities, such as antitumor, anti-inflammatory, antioxidant, and osteogenic effects (Li N et al., 2022; Cariola et al., 2023; Zhang et al., 2016). In recent years, a number of adverse reactions have been associated with psoralens, such as hepatotoxicity, phototoxic dermatitis, and allergy, with hepatic injury being the most common (Shi et al., 2022; Wang et al., 2019). Tian et al. (2017) analyzed 84 cases of adverse reactions due to the use of psoralens from 1978 to 2016. A total of 48 patients had liver injury, which accounted for 57.14% of all cases (Tian et al., 2017). Other clinical studies have shown that PF has a high risk of hepatotoxicity (Li et al., 2019; Rong et al., 2020; Liu et al., 2019). These clinical studies suggest that PF-induced liver injury is mainly hepatocellular injury and cholestasis. The risk of liver injury may increase with an overdose, the use of raw products, or improper dosing.

In the past, PF-induced liver injury has been extensively studied in animal models, such as mice and rats. Wang J et al. (2012) evaluated the effect of FP ethanol extracts (1.875, 1.25, and 0.625 g/kg/day) administered for 28 consecutive days on the liver of Wistar rats, and the results showed that hepatic cholestasis was the main cause of hepatic injury caused by PF. Duan et al. (2020) gavaged male Wistar rats with an FP aqueous extract (2.1 g/kg/day) for 28 consecutive days and found that altered bile acid metabolism and energy metabolism were strongly correlated with hepatic injury via quantitative proteomics and metabolomics analyses. However, some studies have shown that coadministration of PF (0.22 g/kg/day) with Epimedii Folium (EF) for 6 days induces the low-dose lipopolysaccharide-mediated recruitment of hepatic T lymphocytes in rats, possibly leading to specific liver injury. Alanine transaminase (ALT) and aspartate transaminase (AST) levels are significantly elevated, multiple cytokines are overexpressed, and a strong inflammatory response is activated (Gao et al., 2020). The dose and type of liver injury caused by PF, whether inherent or specific, are controversial. Disturbances in bile acid metabolism and transport, oxidative stress, mitochondrial damage, inhibition of hepatocyte regeneration and repair, and inflammatory responses have been the focus of related research (Hou et al., 2020; Wang X et al., 2012; Men et al., 2022). Elucidating the complex mechanism of action of PF is highly challenging because of the problems associated with PF-induced hepatotoxicity. The systematic and efficient study of the process of PF-induced liver injury will help reduce the risk of drug use.

Zebrafish, a new model organism, shares more than 87% homology with humans and is widely used in drug research (Goessling and Sadler, 2015; Cox and Goessling, 2015). Zebrafish have the advantages of small size, easy feeding, high spawning rate, high survival rate, and low reproduction cost, etc. These characteristics can meet the requirements of large sample sizes of experimental animals for toxic drug screening and compensate for the influence of traditional animal models on experimental results due to large individual differences (Rosa et al., 2022; Hernández-Silva et al., 2023). Specific parts of the transgenic zebrafish were stained with fluorescent labels. By observing the location and level of fluorescent markers in specific organs, the target organs affected by drug toxicity can be quickly determined. The safety of 12 kinds of Chinese medicines in Zhuanggu Guanjie pills was rapidly evaluated using a zebrafish model (Chai et al., 2022). Several traditional Chinese medicines (such as *Dipsacus asperata*), which are considered safe, can also cause obvious toxic reactions in zebrafish, but no reports exist on the toxicity of *D. asperata* in traditional animal models. It has been proven that zebrafish are more sensitive to drug toxicity (Cassar et al., 2020). The zebrafish model has the advantages of real-time use, high efficiency, and simplicity in evaluating potential drug toxicity and rapid screening of toxic drugs; it can form a good communication bridge with traditional *in vivo* and *in vitro* models and can be used for preliminary screening of drugs in the early stage of research and development, evaluation of potential toxic components of drugs, and determination of main target organs.

In this study, zebrafish were used to explore the characteristics and mechanism of FP hepatotoxicity. This study provides reliable theoretical support for the use of FP in traditional Chinese medicine and the development and application of preparations.

## Materials and methods

### Plant materials and preparation of FP extracts

FP was purchased from Xinjiang Xinqikang Pharmaceutical Co., Ltd. and authenticated by researcher Shi-xia Huo (Xinjiang Institute of Traditional Uyghur Medicine). Voucher specimens (No. 20190507) were deposited at the Xinjiang Institute of Traditional Uyghur Medicine, China. A measure of 1,000 g of FP was accurately weighed and extracted thrice with 10 volumes of pure water for 1 h (h) by reflux. After filtration, the three aqueous extracts were combined, concentrated, and dried, and a dry powder with a concentration of 7.74 g/g (equivalent to the crude drug) was obtained. The aqueous extract of FP (AEFP) was stored at 2°C–8°C. The main constituents were quantified by HPLC, and AEFP was found to contain psoralen (3.06 mg/g), isopsoralen (2.20 mg/g), and psoralen phenol (14.65 mg/g).

### Chemicals

The FXR agonist obeticholic acid (FXR-A, C10777289, HPLC ≥ 98%) and the peroxisome proliferator-activated receptor α (PPARα) agonist fenofibrate (PPARα-A, C13541890, HPLC ≥ 99%) were purchased from Shanghai Macklin Biochemical Technology Co., Ltd. The FXR inhibitor (Z) guggulsterone (FXR-I, J05j12R136632, HPLC ≥ 98%) and the PPARα inhibitor MK-886 (PPARα-I, C10O11L126310, HPLC ≥ 99%) were purchased from Shanghai Yuanye Biotechnology Co., Ltd. ALT (C009-2-1), AST (C010-2-1), TBA (E003-2-1), TBIL (C019-1-1), γ-GT (C17-2-1), total cholesterol (TC) (A111-1-1), TG (A110-1-1), low-density lipoprotein cholesterol (LDL-C) (A113-1-1), and high-density lipoprotein cholesterol (HDL-C) (A112-1-1) reagent test kits were purchased from Nanjing Jiancheng Bioengineering Institute Co., Ltd.

### Zebrafish maintenance

Zebrafish were reared under standard conditions (14 h of light and 10 h of darkness) in a temperature-controlled (28°C) system. In this study, the transgenic zebrafish Tg (*lfabp:EGFP*) strain was used to label hepatocytes with green fluorescent protein. The zebrafish strains were obtained from the Key Laboratory for Drug Screening Technology of Shandong Academy of Sciences. To obtain the transgenic juvenile fish, healthy and sexually mature female and male fish of the transgenic line lfabp–EGFP were placed in a screened mating box. The barrier was removed at 8:30 a.m. The following morning, zebrafish embryos were obtained from 11:00 to 12:00 a.m. The embryos were washed three times and disinfected with 0.1% methylene blue. The embryos were transferred into zebrafish embryo culture water and cultured at 28°C with 14 h of light control. All the experiments were carried out in compliance with the ethical guidelines and under the supervision of the Ethics Committee of the Biology Institute, Shandong Academy of Sciences.

### Drug treatment

Zebrafish larvae were collected 72 h post-fertilization (hpf). Healthy zebrafish were selected under a microscope and transferred to 6-well plates, with 20 zebrafish in each well. According to the preliminary results, the blank control group (zebrafish culture water), AEFP (40, 50, 60, 70, 80, 90, 100, 110, and 120 μg/mL), FXR-I (1.5 μmol·L^−1^), FXR-I (1.5 μmol·L^−1^ + AEFP 50 μg/mL), FXR-A (5.5 μmol·L^−1^), FXR-A (5.5 μmol·L^−1^ + AEFP 50 μg/mL), PPARα-I (0.5 μmol·L^−1^), PPARα-I (0.5 μmol·L^−1^ + AEFP 50 μg/mL), PPARα-A (6 μmol·L^−1^), and PPARα-A (6 μmol·L^−1^ + AEFP 50 μg/mL) were used, and 200 μM 1-phenyl 2-thiourea (PTU) was added to each group to inhibit melanin production. The mixture was subsequently incubated in a light incubator at a constant temperature of 28.0°C ± 0.5°C for 3 days, after which the solution was changed every day. Three parallel replicates were performed. The development of larvae was observed using an FSX100 Bio Imaging Navigator instrument (Olympus).

### Effect of AEFP on mortality and malformation in zebrafish

The deaths of the zebrafish in each group at 24, 48, and 72 h post-exposure (hpe) were recorded (whether the zebrafish survived was judged by the heartbeat).

### Liver fluorescence area and intensity

Seventy-two hpf zebrafish were anesthetized with tetracaine, and the zebrafish were photographed by fixing their side position (eyes overlapping). The fluorescence area and intensity of the zebrafish liver were observed using an inverted fluorescence microscope. The parameters for fluorescence observation were as follows: excitation wavelength, 490 nm, and emission wavelength, 516 nm (Olympus SZX16, Tokyo, Japan). Image-Pro Plus 5.1 Chinese software was used to measure the area and intensity of liver fluorescence, and GraphPad Prism 6 software was used to construct a histogram for visual comparison.

### Determination of transaminase activity in the zebrafish liver

Seventy-two hours after administration, the zebrafish in the control group and administration group were collected in 1.5-mL EP centrifuge tubes. After 3 rounds of cleaning with 9% normal saline, the cleaning solution was transferred to a preweighed 1.5-mL centrifuge tube after observing that there was no residue. The residual water in the centrifuge tube was removed as much as possible and weighed, and precooled 4-C normal saline was added at a mass ratio of 1:9 (w/w). Juvenile fish (approximately 50 fish) were prepared as 10% tissue homogenates with 180 μL of 4°C normal saline using an ultrasonic crusher. The mixture was centrifuged at 4°C and 3,500 r/min for 10 min, after which the supernatant was collected for later use.

The homogenate was collected to measure the protein concentration of each group by the BCA method, and the enzyme activity and content were subsequently measured according to the instructions of the ALT, AST, TBA, TBIL, γ-GT, TC, TG, LDL-C, and HDL-C kits. The activity of AST and ALT in tissue (U/g prot) = the activity of AST/ALT in the homogenate (U/L) obtained by standard curve ÷ the protein concentration of the homogenate to be measured (g prot/L); TBA, TBIL, γ-GT, TC, TG, LDL-C, and HDL-C content = A (absorbance) to determine ÷ a standard × standard concentration (μmol/L), and the experiment was repeated three times.

### Oil Red O staining

Twenty larvae were randomly selected from each group and fixed in paraformaldehyde at 4°C overnight. On the second day, the larvae were washed with phosphate-buffered saline (PBS) twice and then soaked in PBS containing 25%, 50%, 75%, and 100% propylene glycol for dehydration and infiltration. Then, the sections were dyed with 0.5% Oil Red O solution at room temperature for 4 h. After staining, the cells were gradually rehydrated with PBS and propylene glycol until the larvae were in 100% PBS. Finally, the larvae were fixed on methylcellulose slides.

### Histopathological examination of the zebrafish liver

Ten zebrafish were randomly selected from each group, fixed with 4% paraformaldehyde, dehydrated with an ethanol gradient, and soaked in xylene. Then, the zebrafish were embedded in paraffin, sliced, stained with hematoxylin–eosin (HE), and sealed. The tissue sections were observed and imaged under a microscope (Olympus FSX100, Tokyo, Japan).

### *In situ* hybridization

Partial coding sequences of the zebrafish FXR and PPARα genes were amplified via PCR using first-strand cDNA templates derived from 6 days post-fertilization (dpf) zebrafish juveniles. The forward primer 5′-TCA​AAT​GCC​GTT​GGG​TGG​TA-3′ and reverse primer 5′-TA ATA​CGA​CTC​ACT​ATA​GGG​TGC​AAG​GCT​GTG​AAA​CAA​CAG-3′ were used to amplify the partial PPARα cDNA. The forward primer 5′-TCA​GCT​TGA​CGT​CTT​TTC​CCA-3′ and reverse primer 5′-TAA​TAC​GAC​TCA​CTA​TAG​GGC​ACA​AGT​GAG​CGC​GTT​GTA G-3′ were used to amplify the partial FXR cDNA. PCR products were purified and used as templates for *in vitro* transcription reactions using T7 RNA polymerase (DIGRNALABELINGKIT (SP6/T7) to generate digoxigenin-labeled FXR or PPARα antisense riboprobes. In all the experiments, normal translating ribose was used as a negative control. *In situ* hybridization was performed.

### Analysis of gene expression by RTq-PCR

Fifty zebrafish from each group were homogenized using an ultrasonic pulverizer and added to a 1.5-mL EP tube without enzyme sterilization, after which the RNA was extracted according to the instructions of the SPARK easy IMO proved tissue/cell RNA extraction kit, which was used directly in subsequent experiments.

For RTq-PCR, 2 μL of the synthesized cDNA template was removed, and 10 μL of SYBR qPCR Super Mix Plus was added to a 0.2-mL PCR tube. Then, 1 × 1 μL upstream primer and 1 × 1 μL downstream primer were mixed, and 20 μL of RNase-free water was added. The solution was gently mixed and centrifuged to prepare a 20-μL PCR system with β-actin as the internal reference. The samples were predenatured at 95°C for 60 s, denatured at 95°C for 30 s, annealed at 60°C for 30 s, and annealed at 72°C for 30 s. After 40 cycles, fluorescence quantitative analysis was carried out using PCR software, and CT was obtained. The results of the relative expression of the target gene mRNA were calculated by the 2^−Ct^ method, and the expression multiplier of the target gene in the administration group was calculated by the 2^−Ct^ method. Gene-specific primers for real-time fluorescence quantitative PCR were synthesized by Xi ’an Qingke Biotechnology Co., Ltd., and the primer sequences are shown in Table 1.

**TABLE 1:** The primers used for Real-time quantitative PCR.

Function	Gene		NCBI
Internal reference	β-actin-F	5′-AGA​GCT​ATG​AGC​TGC​CTG​ACG-3′	NC_007112.7
	β-actin-R	5′-CCG​CAA​GAT​TCC​ATA​CCC​A-3′	
Lipid metabolism	PPARγ-F	5′-CAC​TCG​CTG​GAC​ATC​AAG​CC-3′	NC_000005.10
	PPARγ-R	5′-TCC​TGT​AGC​TGT​ACA​TGT​GCG​T-3′	
	PPARαa-F	5′-CGG​GCT​TCA​GGT​TTC​CAC​TA-3′	NC_007136.7
	PPARαa-R	5′-ACG​AAT​AGC​GTT​GTG​GGA​CA-3′	
	SCD1-F	5′-AAC​ACC​AGC​CAA​TCG​GAG​AG-3′	NC_007123.7
	SCD1-R	5′-TGC​TCT​AAA​CAC​GTG​GAC​CC-3′	
	ME1-F	5′-ATG​TTA​CAC​GCA​ACC​CCC​AT-3′	NC_007127.7
	ME1-R	5′-ACC​CGC​AAA​ACT​TGC​ACA​TC-3′	
	ACS-F	5′-CTT​CAG​ACG​CAA​CTT​CCC​CT-3′	NC_007205.1
	ACS-R	5′-CCC​TGT​GGA​AAT​CCT​GCT​GT-3′	
	LPL-F	5′-GCT​CTC​ACG​AGC​GCT​CTA​TT-3′	NC_007133.7
	LPL-R	5′-TCC​TGC​GTG​TGC​GAA​TTT​TG-3′	
	ACS-F	5′-CTT​CAG​ACG​CAA​CTT​CCC​CT-3′	NC_014408.1
	ACS-R	5′-CCC​TGT​GGA​AAT​CCT​GCT​GT-3′	
	CPT1-F	5′-TGC​AGG​GGA​GAT​GTA​GAC​CA-3′	NC_000085.7
	CPT1-R	5′-TGA​CAG​TCC​ACT​TCA​TCG​GC-3′	
	CPT2-F	5′-AAC​TTC​GAG​CAC​TCT​TGG​GG-3′	NC_000001.11
	CPT2-R	5′-GAT​GAG​TCT​ACG​GAC​GCA​GG-3′	
	PGAR-F	5′-CGA​GAT​GAC​ACC​CGA​AGG​AG-3′	NC_000083.7
	PGAR-R	5′-CCG​AGC​CAG​AAC​TCA​CCA​TT-3′	
Bile acid metabolism	FXR-F	5′-GAA​TGA​CCA​CAA​GTT​CAC​C-3′	NC_000003.12
	FXR-R	5′-AAG​AAG​GGA​AGT​CCA​ATA​CC-3′	
	SHP-F	5′-CGA​CTG​TCC​GCT​CAC​TCT​G-3′	NC_007121.7
	SHP-R	5′-CCT​CCT​GCA​GTC​CTG​CTA​TC-3′	
	CYP7A1-F	5′-TTG​CGC​ATG​CTT​TTG​AAC​GA-3′	NC_000008.11
	CYP7A1-R	5′-TCA​AAG​GTT​CGC​CTC​ACC​TC-3′	
	CYP27A1-F	5′-AAC​GCA​TGC​TGC​ATC​CAA​AG-3′	NC_000002.12
	CYP27A1-R	5′-CGC​GTC​TCG​AAG​AGA​ATG​GA-3′	
	CYP8B1-F	5′-CAG​ACG​AAC​CGG​AGA​ACC​TC-3′	NC_000003.12
	CYP8B1-R	5′-CCT​CCG​AGC​TGC​ACT​GTA​AA-3′	
	MRP2-F	5′-GGT​TCA​GGA​GGA​CAT​GTG​GG-3′	NC_054685.1
	MRP2-R	5′-ACC​CTC​AGC​ATC​TAC​GGT​CT-3′	
	BSEP-F	5′-GCA​GGA​CTC​ATG​GCT​CTG​TT-3′	NC_007122.7
	BSEP-R	5′-CCC​CAT​TGT​TGG​GCA​GAG​AT-3′	
	NTCP-F	5′-ATT​GTC​GAG​GCG​CTG​ATC​TT-3′	NC_000002.12
	NTCP-R	5′-TGG​GGC​TCA​TTC​GTC​ACT​TC-3′	

### Statistical analysis

The experimental data were analyzed using SPSS 25.0 software, and the results are expressed as ± S. A *t*-test was used to compare the differences between two groups, and ANOVA was used to compare the differences between multiple groups. GraphPad Prism 6 software was used to construct a graph.

## Results

### Effect of AEFP on the mortality of zebrafish

**FIGURE 1:**
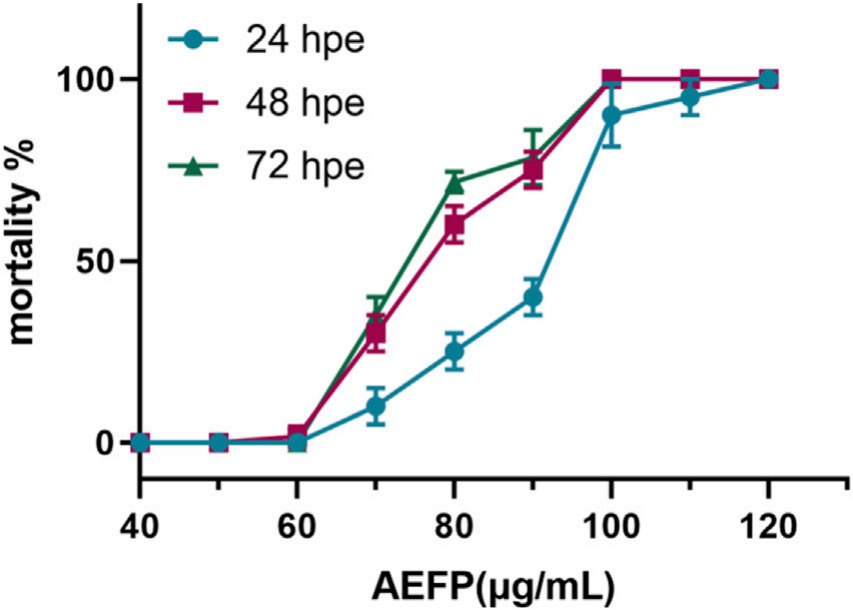
Mortality rate of zebrafish at different concentrations (%).

Using SPSS 21.0, LC_1_ = 55.50 μg/mL, LC_10_ = 63.93 μg/mL, and LC_50_ = 76.04 μg/mL were calculated, as shown in Figure 1. The final concentrations used for drug administration were confirmed to be 40, 50, and 60 μg/mL, as shown in Figure 1.

### Effects of AEFP on zebrafish morphology

**FIGURE 2:**
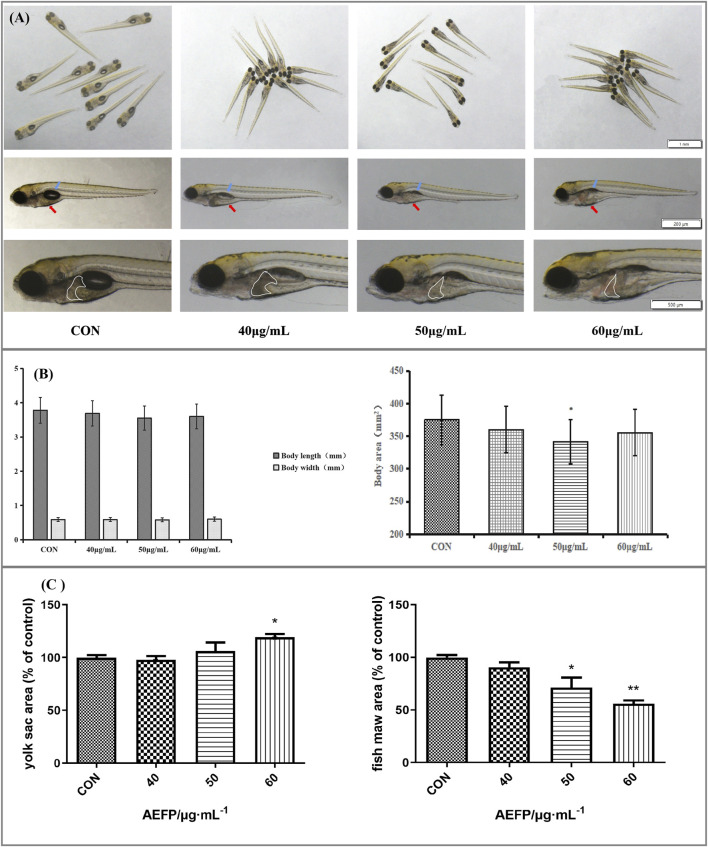
**(A)** Effects of AEFP on swim bladder size, yolk sac absorption, and liver phenotypic changes of zebrafish (blue arrow: swim bladder and red arrow: yolk sac). **(B)** Effects of AEFP on the body length and area growth of zebrafish. **(C)** Effects of AEFP on the yolk sac and fish maw area ( $\bar{x}$ ±s, *n* = 6); * *p* < 0. 05 vs. the CON group.

The morphological changes in the juvenile zebrafish induced by different concentrations of drugs were observed under a fluorescence microscope. The bladders of the zebrafish in the blank control group had a normal shape and clear edges. After 40 μg/mL, 50 μg/mL, and 60 μg/mL AEFP were administered, the swim bladder obviously decreased or even disappeared. The yolk sac absorption of the blank juvenile fish was normal, and the yolk sac absorption of the zebrafish in each administration group was delayed to different degrees, especially in the group administered 60 μg/mL AEFP, as shown in Figures 2A, C. As the concentration of the AEFP administered increased, the body length of the zebrafish had a tendency to decrease, but no significant difference was observed. Moreover, some inhibition of the body area was observed, which was most obvious at 50 μg/mL (*p* < 0.05), as shown in Figure 2B.

### Phenotypic changes in the liver

**FIGURE 3:**
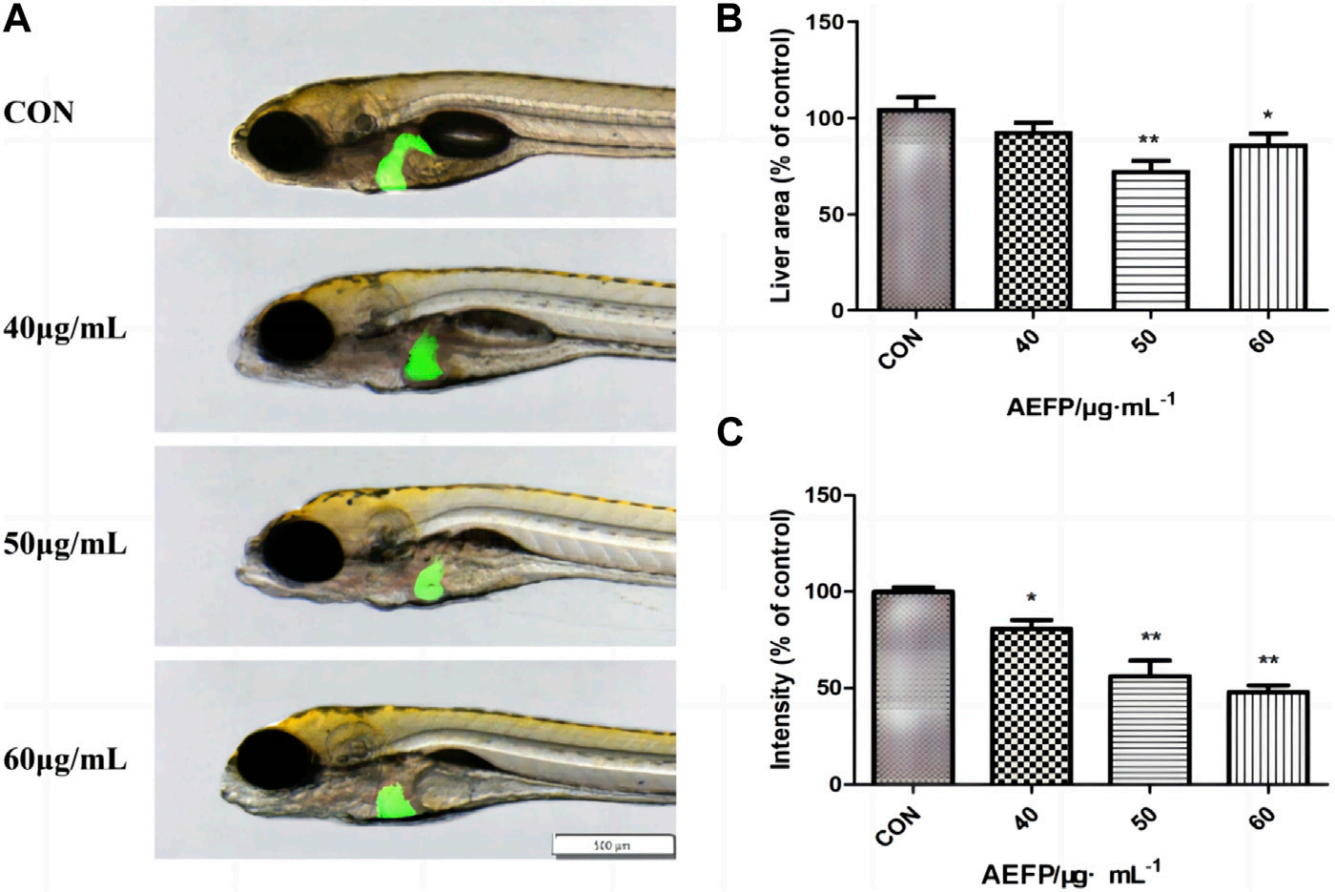
**(A)** Effects of AEFP on liver morphology of Zebrafish; Effects of AEFP on the **(B)** liver fluorescence intensity and **(C)** liver fluorescenc area of zebrafish ( $\bar{x}$ ±s, *n* = 6); *p < 0. 05, and **p < 0.01 vs. the CON group.

Changes in the liver morphology and area of zebrafish after administration were observed under a fluorescence microscope. The livers of the juvenile fish in the blank control group were transparent and normal in shape. The livers of the juvenile fish after treatment with different concentrations of AEFP in a medicated bath showed varying degrees of damage. Compared with that of the blank control group, the liver color of zebrafish treated with 40 μg/mL was gray, and the boundary was unclear. The liver area of the group administered 50 μg/mL AEFP decreased significantly. In the group administered 50 μg/mL AEFP, at 72 hpe, the liver of juvenile zebrafish was obviously atrophied and degenerated, and the liver area was reduced, as shown in Figure 3A.

Compared with those in the blank control group, the fluorescence area and intensity in juvenile zebrafish liver tissue were altered at 72 hpe. In the 50-μg/mL group, the liver obviously atrophied and degenerated, and the fluorescence area decreased. With increasing drug bath concentration, the decrease in liver fluorescence intensity became more obvious and dose-dependent, as shown in Figures 3B, C.

### Effect of AEFP on the biochemical indices of zebrafish

**FIGURE 4:**
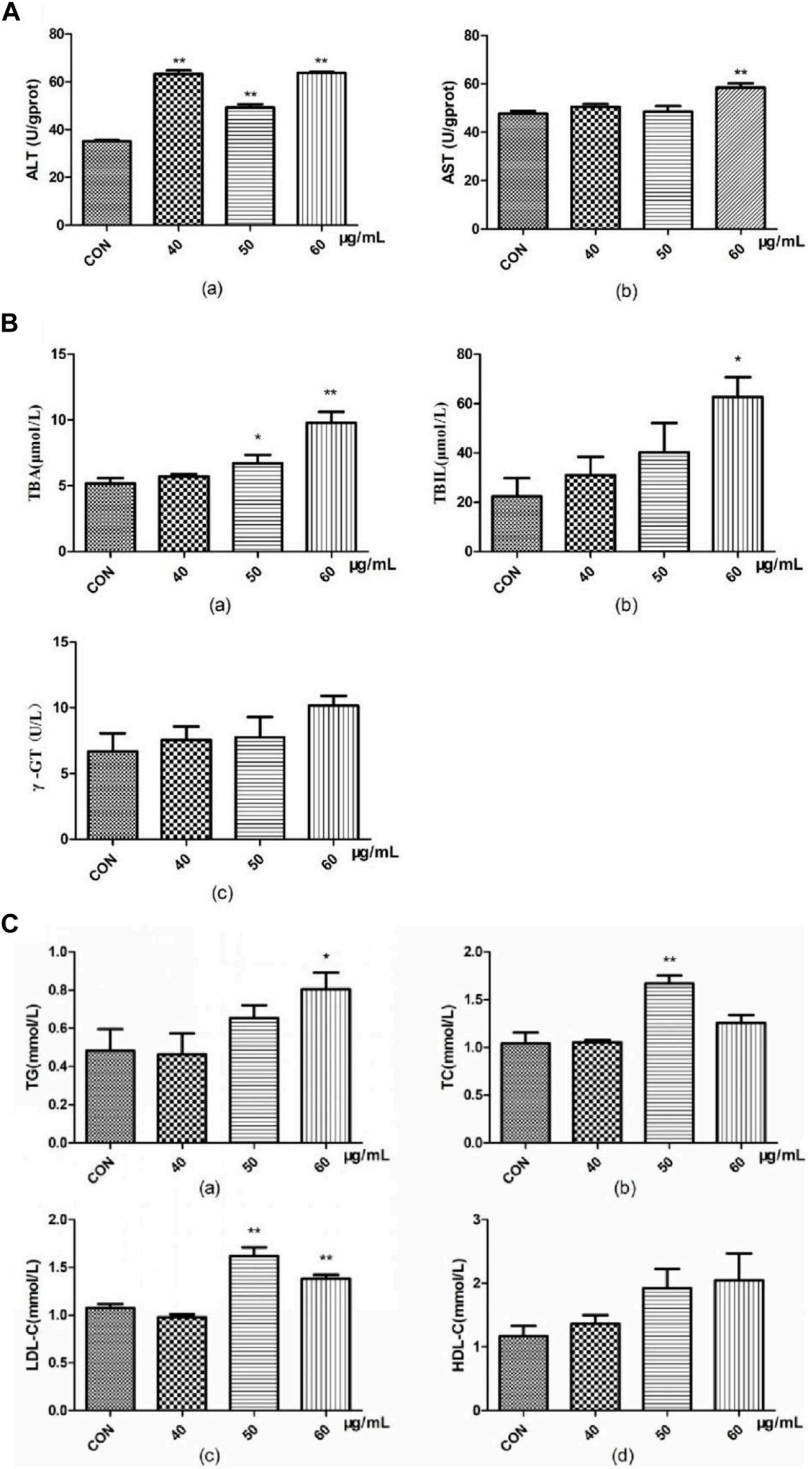
Effects of AEFP on **(A)** liver transaminase, **(B)** bile acid metabolism, and **(C)** lipid metabolism of zebrafish ( $\bar{x}$ ±s, *n* = 6); **p < 0.01 vs. the CON group.

Compared with that in the blank group, ALT activity in the zebrafish in the AEFP group was significantly greater at 72 hpe (*p* < 0.01). At 72 hpe, 60 μg/mL AEFP significantly increased AST activity in zebrafish (*p* < 0.01) (Figure 4A).

Compared with those in the blank group, the TBA content in the groups administered 50 μg/mL and 60 μg/mL AEFP significantly increased at 72 hpe (*p* < 0.05 and *p* < 0.01, respectively). At 72 hpe, treatment with 60 μg/mL AEFP significantly increased the TBIL concentration in the zebrafish (*p* < 0.05) (Figure 4B).

Compared with those in the blank group, the doses of 50 and 60 μg/mL AEFP significantly increased the TC content in zebrafish at 72 hpe (*p* < 0.05). At 72 hpe, a dose of 50 μg/mL AEFP significantly increased the TC content in the zebrafish (*p* < 0.05). Concentrations of 50 μg/mL and 60 μg/mL significantly increased the LDL-C concentration (*p* < 0.05) (Figure 4C).

### Observation of lipid deposition in zebrafish liver tissue

**FIGURE 5:**
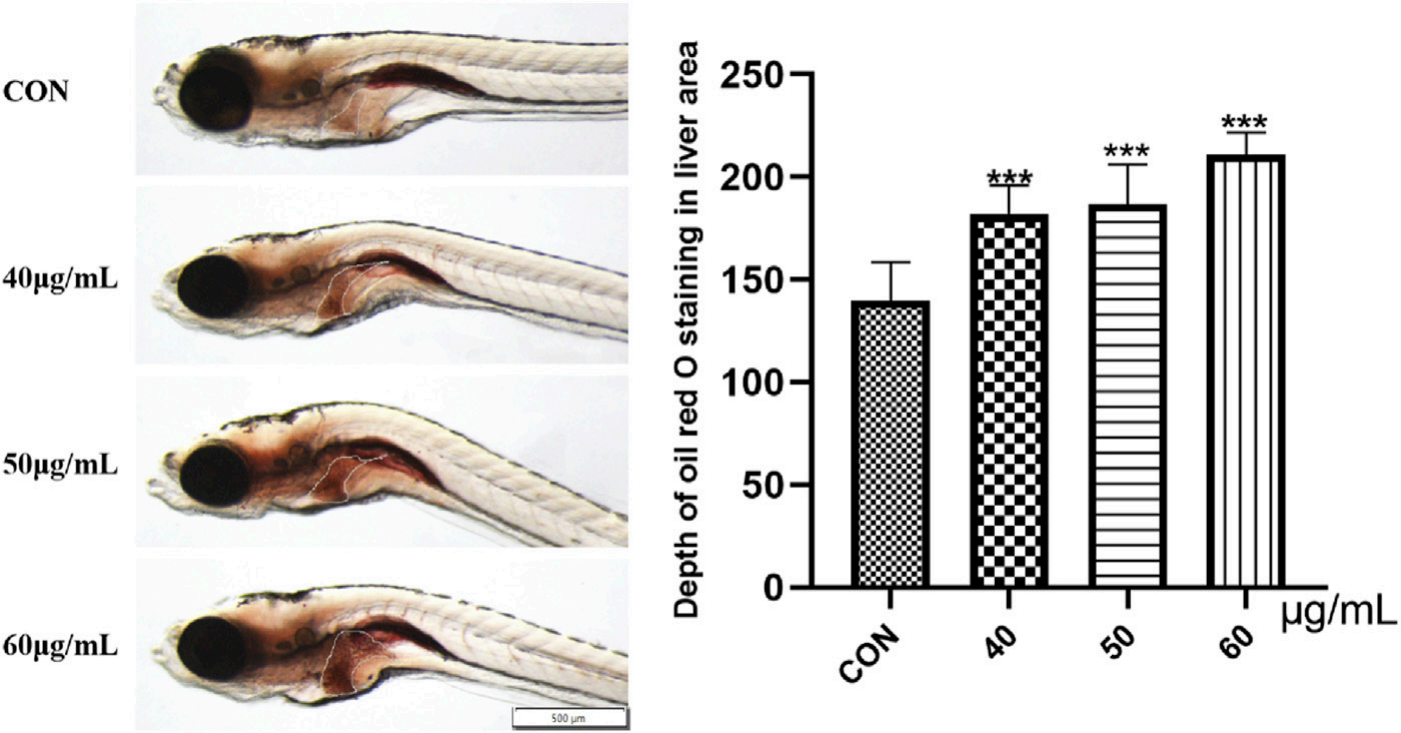
Effects of AEFP on lipid deposition in zebrafish. ***p < 0.001 vs. the CON group.

Oil Red O staining was used to study the accumulation of fat in the zebrafish liver. As shown in Figure 9, compared with that in the blank control group, the liver Oil Red O staining in the 40-μg/mL, 50-μg/mL, and 60-μg/mL AEFP dosage groups was deepened to different degrees after 72 hpe was administered, and the color gradually deepened with increasing dosage (*p* < 0.001). This finding indicates that AEFP can lead to fat deposition in zebrafish liver tissue, as shown in Figure 5.

### Histopathological observation of liver tissue

**FIGURE 6:**
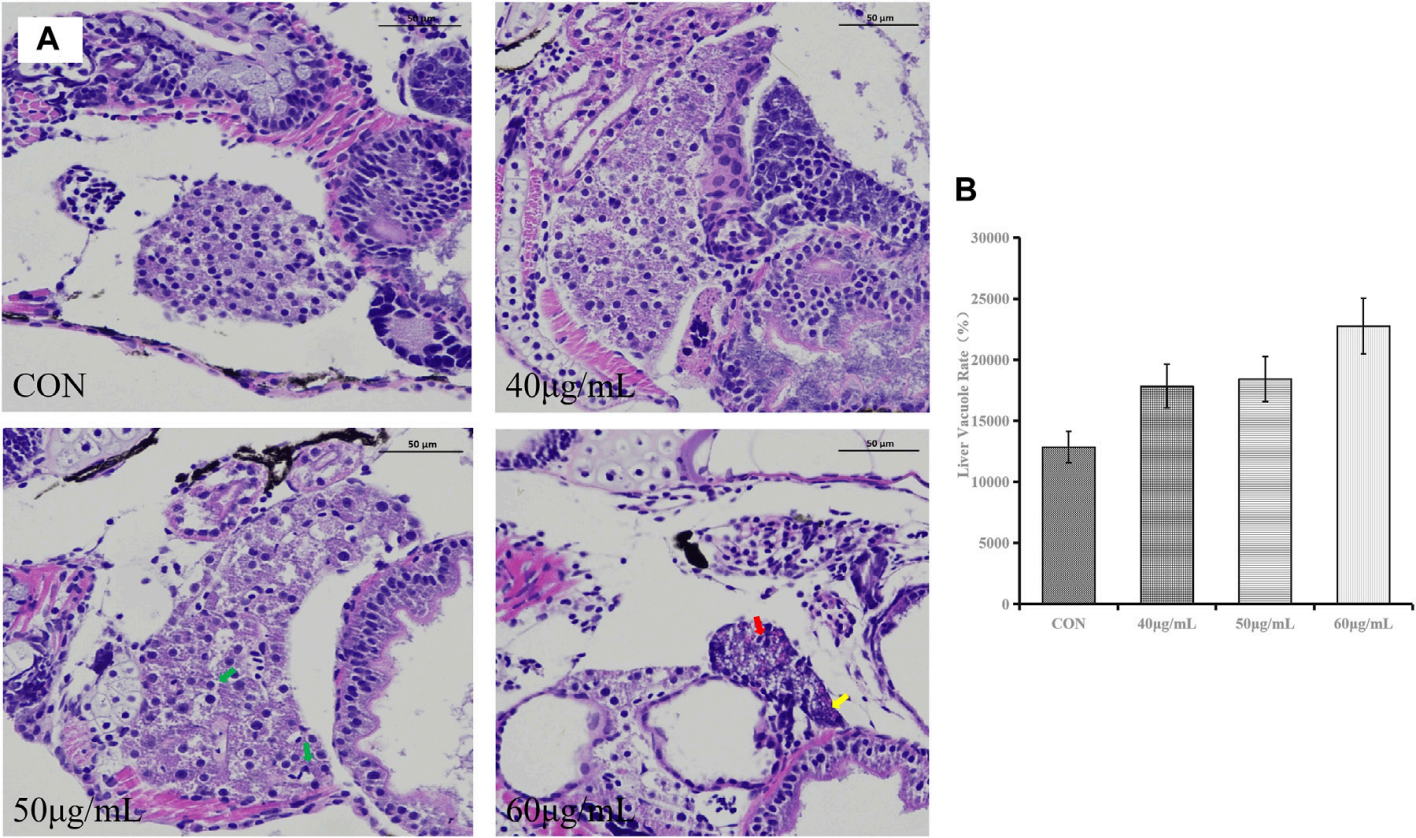
**(A)** Typical histopathological section photographs of zebrafish liver specimens for HE analysis (magnification ×200 and ×100); green arrow: hepatocyte vacuolation, yellow arrow: nuclear pyknosis, and red arrow: steatosis. **(B)** Effects of AEFP on the liver vacuole rate in zebrafish ( $\bar{x}$ ±s, *n* = 6), vs. the CON group.

In the control group, the liver structure was normal, with clear cell margins and close contact. In the group treated with 40 μg/mL AEFP, no obvious histological change was observed except for the loose connection of local hepatocytes. Compared with those in the control group, some hepatocytes in the group treated with 50 μg/mL AEFP were vacuolated. In the group treated with 60 μg/mL AEFP, the morphology of the hepatocytes was irregular, the volume decreased, and fatty degeneration of the hepatocytes occurred, as shown in Figure 6A. The results given in Figure 6B show that AEFP induced an increase in the rate of vacuolation in the zebrafish liver, but no significant trend was observed.

### Effect of AEFP on the mRNA expression in zebrafish

**FIGURE 7:**
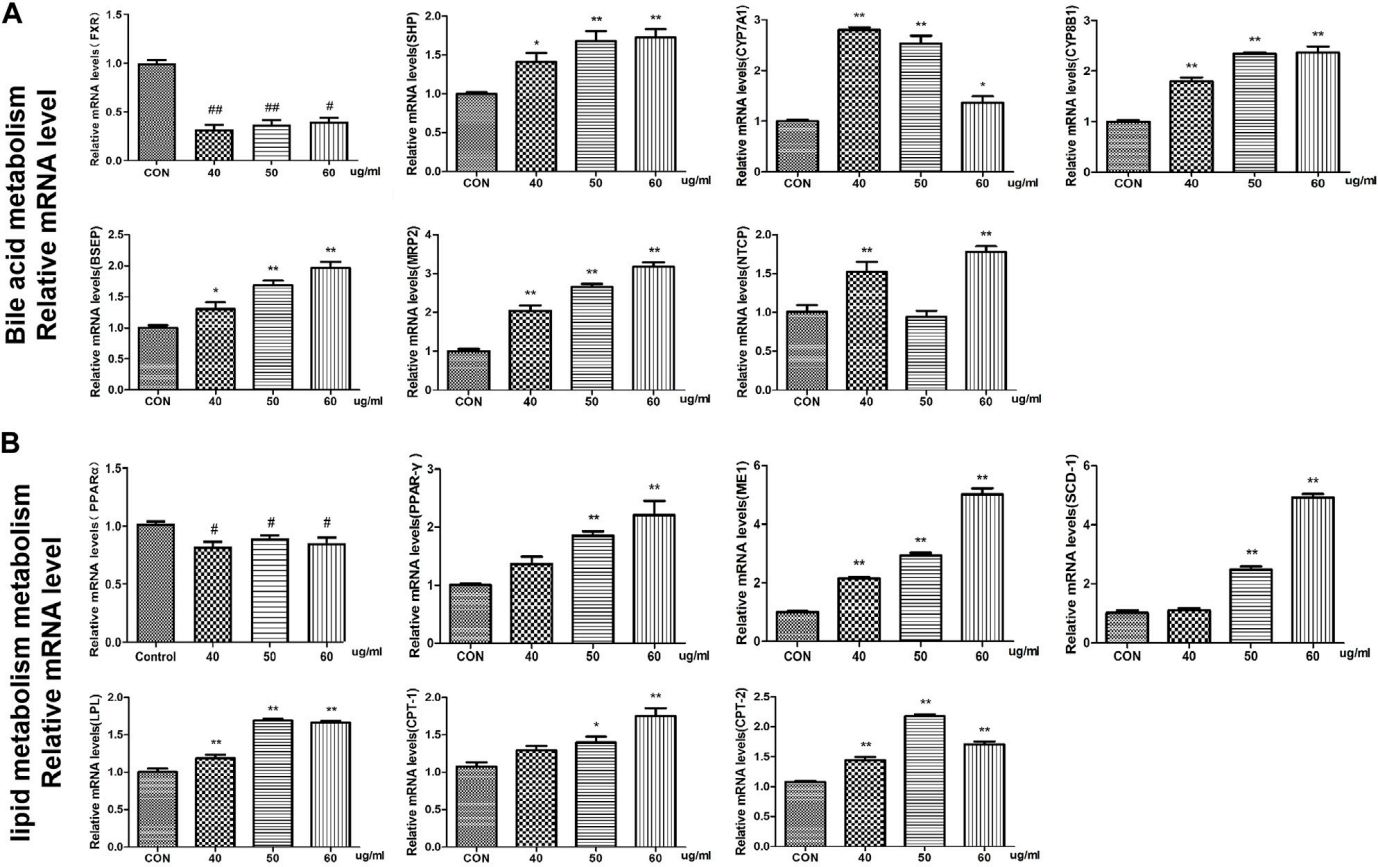
Effects of WEFP on the expression of liver injury-related genes in zebrafish **(A)**. Gene expression of the bile acid metabolism pathway. **(B)** Influence of key genes in the fat metabolism pathway;*p < 0. 05 and **p < 0. 01 vs. the CON group.

At the mRNA level, we found that the expression of the FXR and CYP27A1 genes was inhibited in the bile acid metabolism signaling pathway in the AEFP-treated group, whereas the expression levels of SHP, CYP7A1, CYP8B1, BSEP, MRP2, and NTCP were significantly increased at 72 hpe after the administration of AEFP (aqueous extract of FP) (*p* < 0.01). WEFP also had a regulatory effect on key genes involved in lipid metabolism, i.e., PPARα, peroxisome proliferator-activated receptor γ (PPARγ), ME-1, SCD-1, lipoprotein lipase (LPL), CPT-1, CPT-2, and PGAR. The regulatory effect and the expression levels of PPARα, PPARγ, ME-1, SCD-1, LPL, CPT-1, CPT-2, and PGAR were significantly greater than those in the blank control group at 72 hpe after drug administration (*p* < 0.01), as shown in Figure 7.

### Effects of the combination of AEFP with FXR and PPARα agonists and inhibitors on the liver

**FIGURE 8:**
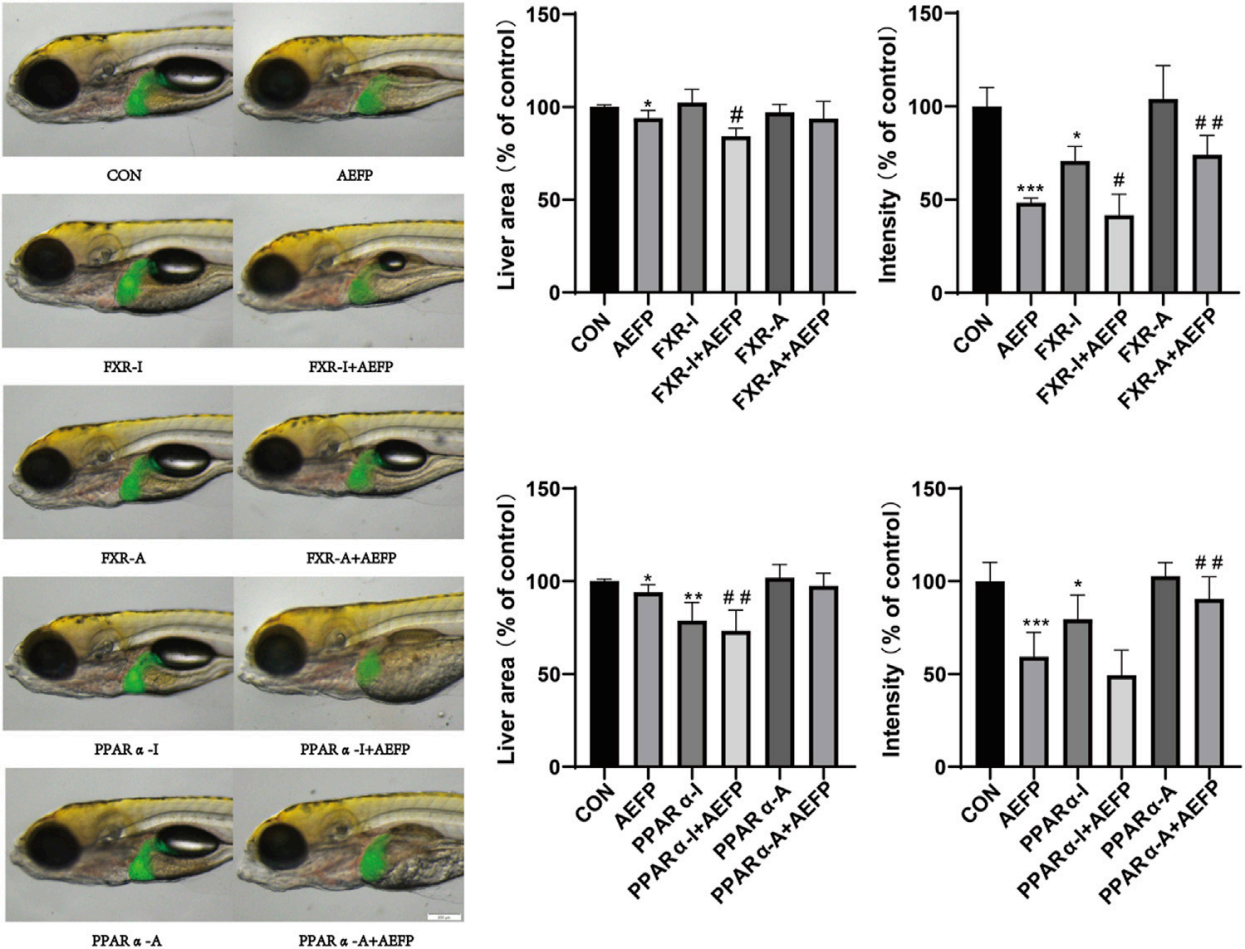
Effects of AEFP combined with FXR and PPARα agonists and inhibitors on the liver fluorescence intensity and area of zebrafish. The concentration of AEFP is 50 μg/mL, FXR-I is 1.5 μM, FXR-A is 5.5 μM, PPARα-I is 0.5 μM, and PPARα-A is 6 μM. *p < 0. 05 and ***p < 0.001 vs. the CON group; ^#^ *p* < 0. 05 and ^##^ *p* < 0.01 vs. the AEFP group.

Compared with that in the control group, the liver area in the AEFP-alone group was significantly lower (*p* < 0.05), and the liver fluorescence intensity was significantly lower (*p* < 0.001); moreover, the liver fluorescence intensity in the FXR inhibitor-alone group was significantly lower (*p* < 0.05, *p* < 0.001). The liver area in the PPARα inhibitor group was significantly reduced (*p* < 0.01), and the fluorescence intensity in the liver was significantly decreased (*p* < 0.05). Compared with that in the AEFP group, the liver area in the FXR-A and AEFP groups was significantly lower (*p* < 0.05), and the liver fluorescence intensity was significantly lower (*p* < 0.05). In the FXR stimulation group, the liver area in the zebrafish coadministered with the PPARα inhibitor and AEFP increased significantly (*p* < 0.05), and the liver fluorescence intensity increased significantly (*p* < 0.01). In the group coadministered with the PAPRα inhibitor and AEFP, the liver area decreased significantly (*p* < 0.01), and the liver fluorescence intensity tended to weaken. In the group administered with the PPARα agonist and AEFP together, the liver area increased, and the liver fluorescence intensity increased significantly (*p* < 0.01) (Figure 8).

### Effects of FXR and PPARα agonist inhibitors on the expression of FXR and PPARα in the livers of zebrafish

**FIGURE 9:**
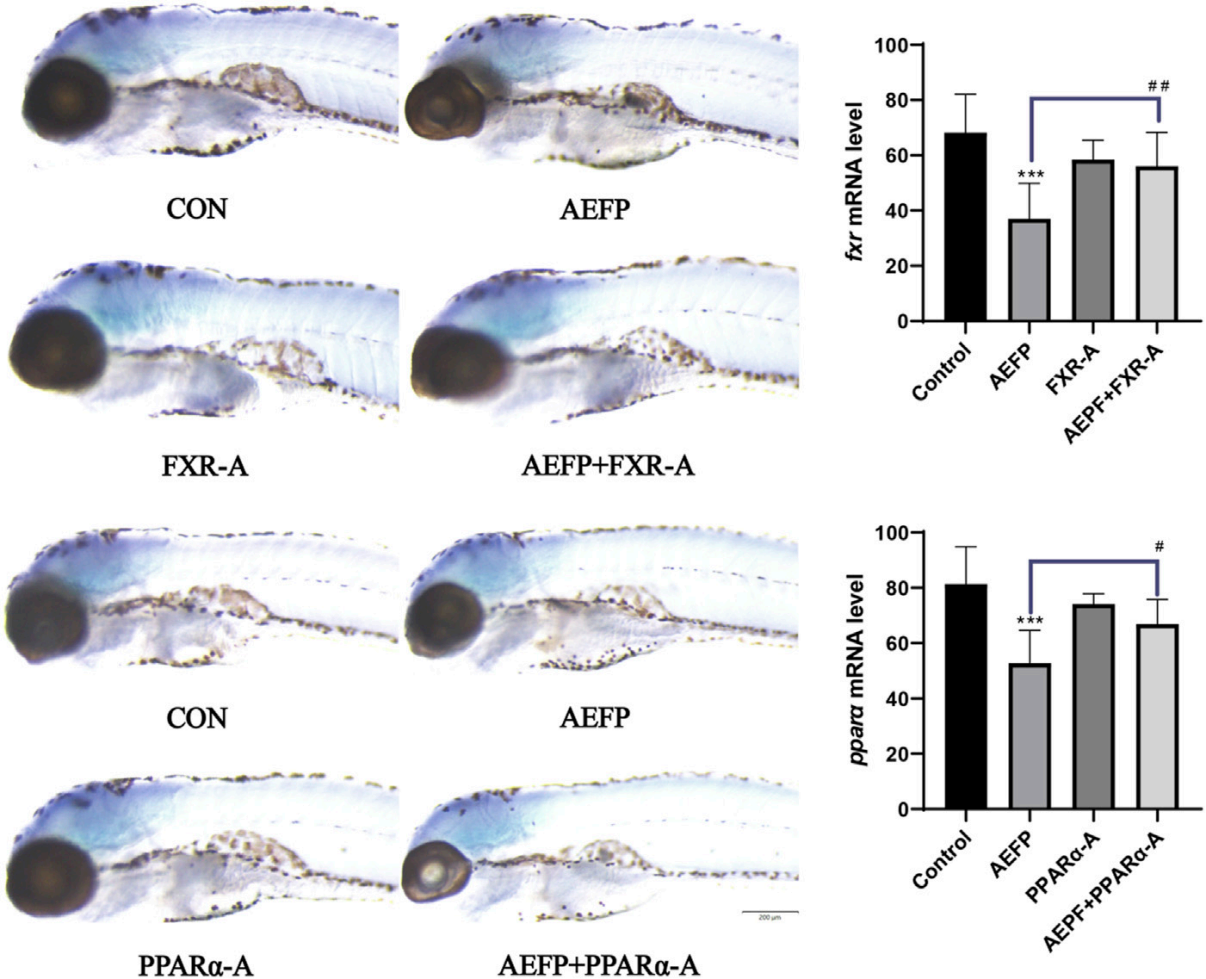
Effect of combined administration on the results of *in situ* hybridization. The concentration of AEFP is 50 μg/mL, FXR-I is 1.5 μM, FXR-A is 5.5 μM, PPARα-I is 0.5 μM, and PPARα-A is 6 μM. ***p < 0.001 vs. the CON group; ^#^ *p* < 0. 05 and ^##^ *p* < 0.01 vs. the AEFP group.

Compared with those in the control group, the staining depth of FXR and PPARα in the liver in the AEFP group was decreased, the FXR staining depth in the liver in the FXR agonist group was increased, and the depth of PPARα staining in the liver in the PPARα agonist group was increased. Compared with that in the AEFP-alone group, the depth of liver FXR staining in the FXR agonist and AEFP coadministration group was greater; moreover, the depth of liver PPARα staining in the PAPRα agonist and AEFP coadministration group was greater, as shown in Figure 9.

### Effects of AEFP combined with FXR and PPARα agonists and inhibitors on the mRNA expression of key genes involved in bile acid metabolism and lipid metabolism pathways

The mRNA levels of FXR, SHP, and PPARα in the AEFP group were significantly lower (*p* < 0.05) than those in the control group, while the mRNA levels of CYP7A1 and LPL were significantly greater (*p* < 0.05). In the FXR inhibitor group, the mRNA levels of FXR and SHP were significantly lower (*p* < 0.05), while the mRNA level of CYP7A1 was significantly greater (*p* < 0.001). In the FXR agonist group, the mRNA levels of FXR and SHP were significantly increased (*p* < 0.05), while the mRNA level of CYP7A1 was significantly decreased (*p* < 0.001). In the PPARα inhibitor group, the mRNA level of PPARα was significantly decreased (*p* < 0.001), and the mRNA level of CYP7A1 was significantly decreased (*p* < 0.05). The mRNA levels of PPARα and LPL were significantly lower in the PPARα agonist group (*p* < 0.01).

**FIGURE 10:**
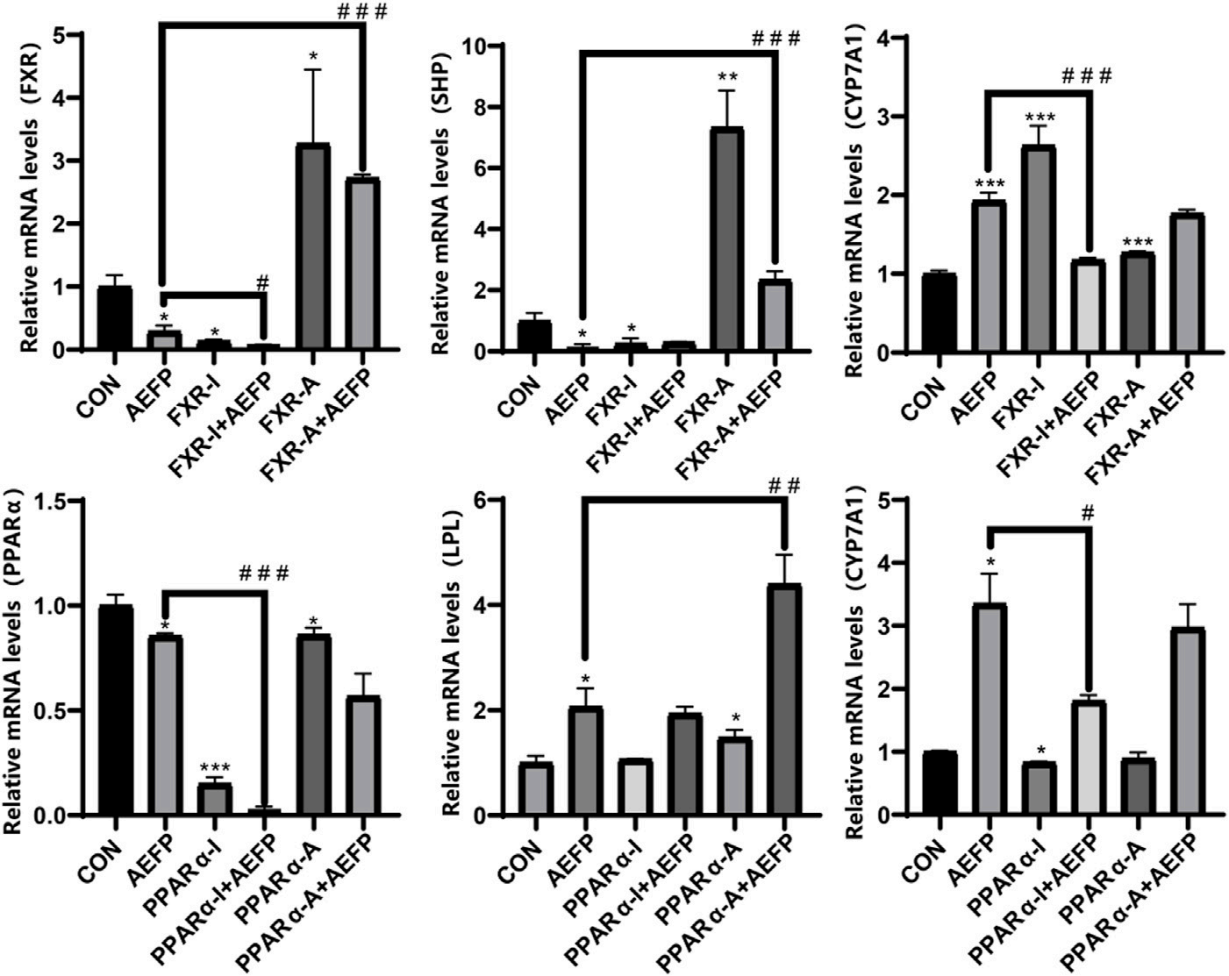
Effect of combined administration on the results of mRNA. The concentration of AEFP is 50 μg/mL, FXR-I is 1.5 μM, FXR-A is 5.5 μM, PPARα-I is 0.5 μM, and PPARα-A is 6 μM. *p < 0.05, **p < 0.01, and ***p < 0.001 vs. the CON group; ^#^ *p* < 0. 05, ^##^ *p* < 0.01, and ^###^3 *p* < 0.001 vs. the AEFP group.

Compared with those in the AEFP group, the mRNA levels of FXR were significantly downregulated (*p* < 0.05), and the mRNA level of CYP7A1 was significantly downregulated (*p* < 0.001) in the coadministration group of the FXR inhibitor and AEFP. The mRNA levels of FXR and SHP in the coadministration group of the FXR agonist and AEFP were significantly upregulated (*p* < 0.001); the mRNA levels of PPARα in the coadministration group of the PAPRα inhibitor and AEFP were significantly decreased (*p* < 0.001), and the mRNA level of CYP7A1 was significantly downregulated (*p* < 0.05); and the mRNA level of LPL in the coadministration group of the PAPRα agonist and AEFP was significantly upregulated (*p* < 0.01), as shown in Figure 10.

## Discussion

The use of zebrafish is an important tool for high-throughput screening of drug hepatotoxicity. Although the liver structure of zebrafish is different from that of mammals, the basic physiological processes, genetic mutations, and pathogenic responses of zebrafish to environmental damage are highly similar (Bala et al., 2020; Bambino et al., 2019). In this study, transgenic lfabp:EGFP zebrafish were used as experimental models. After being exposed to different concentrations of AEFP for 72 hpe, the liver was damaged to different degrees, as indicated by a gray color, blurred edges, and a reduced area compared with those in the blank group. With increasing drug concentration, the color of the liver gradually deepened. Moreover, the fluorescence area and intensity in the liver decreased to different degrees. Further observation showed that, compared with that in the blank group, the delay in yolk sac absorption was most obvious when 60 μg/mL AEFP was administered. Approximately 70% of the yolk sac of zebrafish is composed of neutral lipids, which provide nutrients during early embryonic development (Katoch and Patial, 2021; Zhang et al., 2019; Jiang et al., 2022). When the liver is damaged, the metabolic rate of the yolk sac slows, hindering lipid metabolism in the yolk sac and thus causing an absorption delay in the yolk sac. Yolk sac absorption can also be used as an indirect indicator of liver function (Bambino et al., 2019).

The liver biomarkers ALT and AST are important indices for evaluating liver function and mainly exist in the cytoplasm of hepatocytes. Damage to hepatocytes leads to an increase in cell membrane permeability, the release of ALT and AST into the body, and an increase in transaminase levels (Lala et al., 2023; Zhang et al., 2023). After AEFP treatment, the levels of ALT and AST in the zebrafish increased significantly, indicating that AEFP has a certain toxic effect on zebrafish hepatocytes. Moreover, after AEFP administration, the total bile acid, total bilirubin, total cholesterol, triglyceride, LDL-C, and HDL-C levels in zebrafish increased, suggesting that AEFP can cause cholestasis and lipid metabolism disorders. In addition, the liver phenotype, Oil Red O staining, and pathology results showed that the fluorescence intensity of the liver decreased obviously after administration at 72 hpe, and lipid deposition was observed in the liver. The hepatotoxic effect of AEFP on zebrafish was confirmed, and it was hypothesized that the mechanism of hepatotoxicity of AEFP might be related to hepatocellular injury, cholestasis, and lipid metabolism disorders.

Therefore, the study of pathways associated with hepatic bile acid and lipid metabolism is an important direction for elucidating the mechanisms of AEFP hepatotoxicity. Altered expression of genes related to cholesterol biosynthesis and bile acid metabolism was revealed by mRNA analysis of zebrafish liver samples. Bile formation is an essential function of the liver, and bile acids (BAs), which are evolutionarily conserved molecules synthesized from cholesterol in the liver, are critical for the regulation of bile metabolism and lipid metabolism (Chiang and Ferrell, 2018). The lipoid X receptor (FXR) was the first receptor demonstrated to be activated by endogenous bile acids, and FXR plays a crucial role in the regulation of bile acid homeostasis. FXR inhibits the expression of genes related to bile acid synthesis (CYP7A1 and CYP8B1) and, thus, reduces the bile acid concentration in hepatocytes (Appelman et al., 2021; Lee et al., 2021). In addition, FXR activation limits bile acid accumulation in hepatocytes by inhibiting the expression of the bile acid membrane transporter protein NTCP and induces bile acid efflux from the liver by upregulating the expression of the bile acid efflux pump BSEP. The FXR/SHP pathway controls the homeostasis of cholesterol and bile acids in enterohepatic circulation, and FXR inhibits SHP transcription when bile acid levels are elevated (Yu et al., 2021). The results showed that AEFP inhibited the expression of FXR in the liver and that feedback induced the expression of the SHP, CYP7A1, and CYP8B1 genes, thus increasing the synthesis of bile acids; moreover, it induced the upregulation of NTCP expression, causing the accumulation of bile acids in hepatocytes. The increase in the expression of the BSEP and MRP2 genes may be a compensatory response caused by cholestasis. Hepatic transporters play a crucial role in the (ATP-dependent) efflux of BAs and substrates into somatic circulation. MRP2, a member of the multidrug resistance-associated gene family, is expressed in the basolateral membranes of hepatocytes and undergoes adaptive upregulation in response to bile deposition injury or BA feeding (Wang et al., 2023). Additionally, FXR is an important regulator of lipid metabolism. Studies have shown that the bile acid-induced FXR/SHP pathway reduces TG levels by inhibiting adipogenic sterol regulatory element-binding protein 1 (SREBP-1c), leading to the repression of genes involved in adipogenesis, including desaturase 1 (SCD) and ME-1 (Ding et al., 2021). Another study showed that FXR could prevent hepatic TG accumulation by inducing PPARα activity and stimulating fatty acid β-oxidation in human hepatocellular carcinoma cells (Guan et al., 2022).

Moreover, PPARα efficiently induces the expression of numerous genes involved in a variety of lipid metabolic pathways, including microsomal, peroxisomal, and mitochondrial fatty acid oxidation; fatty acid doping and activation; fatty acid elongation and desaturation; triglyceride synthesis and catabolism; lipoprotein metabolism; glucose metabolism; bile acid metabolism; and a wide range of other metabolic pathways and genes (Samuel and Shulman, 2018; Kersten and Stienstra, 2017). Thus, PPARα deficiency or suppressed expression can cause a reduction in the transcript levels of a series of proteins and enzymes related to fatty acid metabolism in the liver, leading to reduced fatty acid oxidation, impaired lipoprotein anabolism, and intracellular fat deposition in the liver (Attema et al., 2022; Zeng et al., 2022; Yu et al., 2015). PPARγ and LPL are both genes that promote lipolysis. Activation of PPARγ reduces fatty acid delivery to the liver and muscle and decreases fat synthesis. Moreover, PPARγ induces the expression of LPL in adipocytes, which promotes lipid metabolism and reduces blood lipid levels, thereby increasing plasma HDL, LDL, and TG levels. The enzyme LPL is one of the key factors in the process of adipogenic differentiation [Nakamura et al., 2014; Lim et al., 2021].

Moreover, we confirmed that when FXR and PPARα agonists were coadministered with AEFP, the original decreases in fluorescence intensity and area indices in the livers of the zebrafish caused by AEFP were reversed. In contrast, the administration of FXR and PPARα inhibitors reduced the fluorescence intensity and area. The decrease intensified. After testing the relevant mRNA levels (SHP, CYP7A1, and LPL), it was found that AEFP may cause hepatotoxicity by downregulating the expression of the FXR and PPARα genes and affecting downstream pathways. ISH further confirmed that the regulation of the FXR and PPARα genes by AEFP is concentrated in the liver.

**FIGURE 11:**
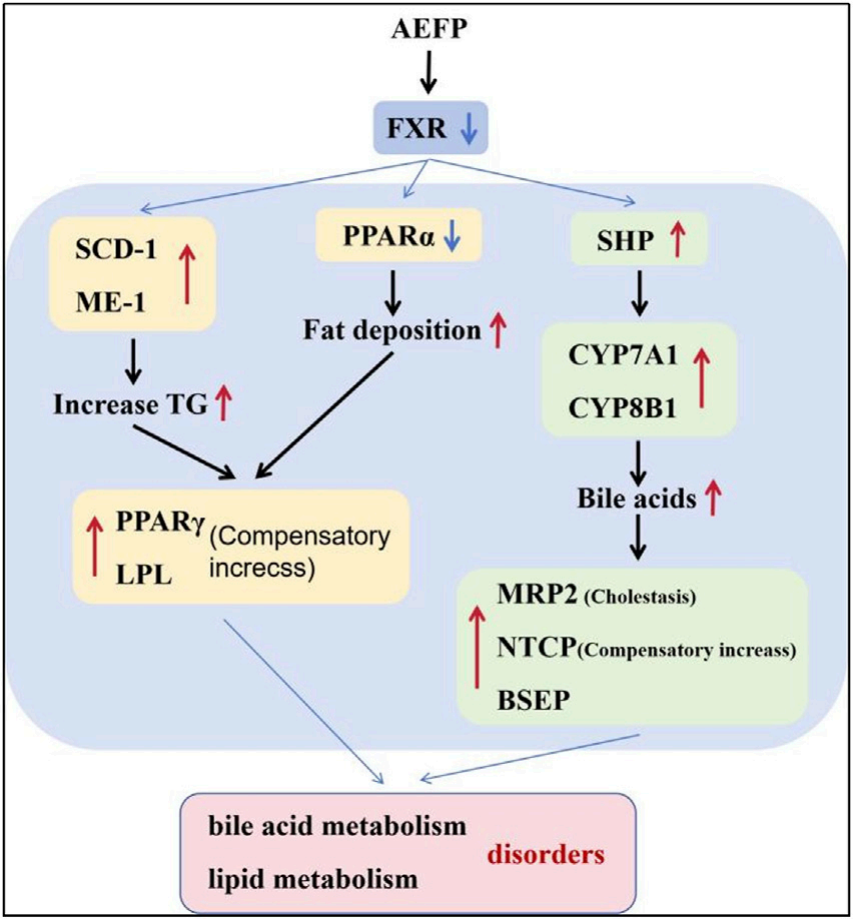
Effects of AEFP on the pathways in zebrafish.

AEFP can cause liver injury in juvenile zebrafish via liver inflammation and lipid metabolism disorders, which leads to fat deposition by activating the inflammasomes and inhibiting the expression of key target genes of the PPAR signaling pathway. FXR expression was inhibited, which caused cholestasis and further aggravated the occurrence of liver injury. The AEFP pathway in zebrafish is shown in Figure 11.

## Data Availability

The datasets presented in this study can be found in online repositories. The names of the repository/repositories and accession number(s) can be found in the article/Supplementary Material.
